# Physical Demands in Elite Futsal Referees During Spanish Futsal Cup

**DOI:** 10.3389/fpsyg.2021.625154

**Published:** 2021-01-22

**Authors:** Carlos Serrano, Javier Sánchez-Sánchez, Jose Luis Felipe, Enrique Hernando, Leonor Gallardo, Jorge Garcia-Unanue

**Affiliations:** ^1^IGOID Research Group, Department of Physical Activity and Sport Sciences, University of Castilla-La Mancha, Toledo, Spain; ^2^School of Sport Sciences, Universidad Europea de Madrid, Madrid, Spain

**Keywords:** match monitoring, activity profile, indoor tracking system, physical performance, futsal

## Abstract

In futsal there are two referees on the playing court and their capacity to respond to physical and physiological demands imposed during the game is essential for the success. The futsal characteristics such as size pitch, referees position and rules of games or type of league could impose specific physical efforts probably. The aim of this study were to analyze the physical demands of eight elite referees (age 40 ± 3.43 years; height 1.80 ± 0.03 m; weight 72.84 ± 4.01 kg) from seven matches of Spanish Futsal Cup 2020. The physical activity of each referee during the match was monitored with a Local Positioning System, which was installed on futsal pitch where the matches were played. The data differences were evaluated as Paired-Samples *T*-Test procedure. The results revealed a similar total distance between halves (2888.39 vs. 2831.51 m). The zone 3 distance (15.1–18 km⋅h^–1^) showed a significative decrease (*p* < 0.05) during the match in comparison to the first and second halves (−24.48 m; CI95%: −9.54 to −39.42; ES: 0.56). The number of high-intensity acceleration (−10.29; CI95%; 3.71–16.86; ES: 0.92) and deceleration (−24.86; CI95%; 11.59–38.12; ES: 0.99) decreased in the second half of the match (*p* < 0.05). Therefore, the use of the tracking device to monitoring physical performance provides knowledge of the specific activity profile from futsal referees. This information to can be useful to design more accurate the training programs.

## Introduction

Team sports have a referee, who has the responsibility of ensuring matches develop according to the regulations. For this reason, they must follow the game with full attention and precision control (Borin et al., 2013). Additionally, the referee’s capacity to respond to physical and physiological demands imposed during the game is essential for the success of refereeing in different sports (Reilly and Gregson, 2006; Weston et al., 2007; Bloß et al., 2020; García-Santos et al., 2020). The physical demands of referees have been the subject of studies during the last years in different team sports like Australian football (Elsworthy et al., 2014), rugby (Brightmore et al., 2016), or football (Castagna et al., 2019) due to the development of wearable technology, such as global positioning systems (Chambers et al., 2015; Scott et al., 2016).

Futsal is an indoor team sport with a 2 × 20 min game of high-intensity and intermittent actions (Naser et al., 2017). The specific characteristics of futsal, such as the dimension of pitch, position of the referee, or rules of the game, may lead to different physical and physiological efforts to football, in spite of the similarities of refereeing in these two sports (Rebelo et al., 2011). The recent progress in technology has made it possible to track time and motion analysis and the physical demands of indoor team sports with validity and accuracy in reference to determining distance covered, speed, mean velocity, accelerations, and decelerations for intermittent activities (Bastida Castillo et al., 2017; Vieira et al., 2017; Serpiello et al., 2018; Bastida-Castillo et al., 2019; Gómez-Carmona et al., 2019). Regarding indoor team sports, referees’ match performance has been analyzed in futsal with video analysis (Rebelo et al., 2011) and basketball with tracking devices (Borin et al., 2013; García-Santos et al., 2019; Leicht et al., 2019).

In futsal there are two principals referees on the playing court and it is necessary that they maintain a good position regarding the run of play in the game in order to observe possible infractions. In reference to these requirements, Rebelo et al. (2011) showed the activity of futsal referees is generally characterized by intermittent moderate to high intensity running with numerous very brief bouts of fast speed and sprint, interspersed by long periods of low-intensity recovery. Moreover, there are a lot of high neuromuscular actions required due to large demands of backward movements (Ahmed et al., 2017).

In the last few years, the inertial devices with Ultra-Wide Band (UWB) technology by local positioning systems (LPS) have enabled the monitoring of positioning and the obtaining of the performance of elite futsal players, it showed the activity profile during official competition (Illa et al., 2020; Ribeiro et al., 2020; Serrano et al., 2020). Nevertheless, the scientific knowledge regarding the physical and physiological demands of professional referees during competition exist, further evidences are necessary to establish an accurate activity profile. Additionally, there are not previous information of physical demands or movement patterns of elite futsal referees with tracking technology device during official games, it was only with video-analysis systems (Rebelo et al., 2011; Ahmed et al., 2017).

Therefore, the aim of the present manuscript was to analyze the activity profile and to compare the physical demands of elite futsal referees between first and second halves during official matches from Spanish Futsal Cup using Local Positioning System technology for monitoring their movement patterns.

## Materials and Methods

### Participants

A total of eight elite Spanish futsal referees (age 40 ± 3.43 years; height 1.80 ± 0.03 m; weight 72.84 ± 4.01 kg) participated in this study (14 observations, seven matches and two referees per match). The power of the statistical results was 0.9196 for the selected sample. The training program of referees was constituted by five sessions per week. Training sessions were composed of aerobic-anaerobic work and injury prevention exercises. All of them had at least 10 years of experience in the first division of the National Spanish Futsal League (LNFS). They were selected by the National Committee of Referees for participating in the Spanish Futsal Cup 2020.

### Design

The referees were monitored during seven games, which were distributed in the quarter-finals, semi-finals and final over 4 days. All of the participants were informed about the study requirements and provided written informed consent. The study protocol was approved and followed the guidelines established by the local Institution – Ethics Committee of the European University of Madrid (CIPI35/2019) – and in accordance with the recommendations of the Declaration of Helsinki.

### Methodology

**FIGURE 1:**
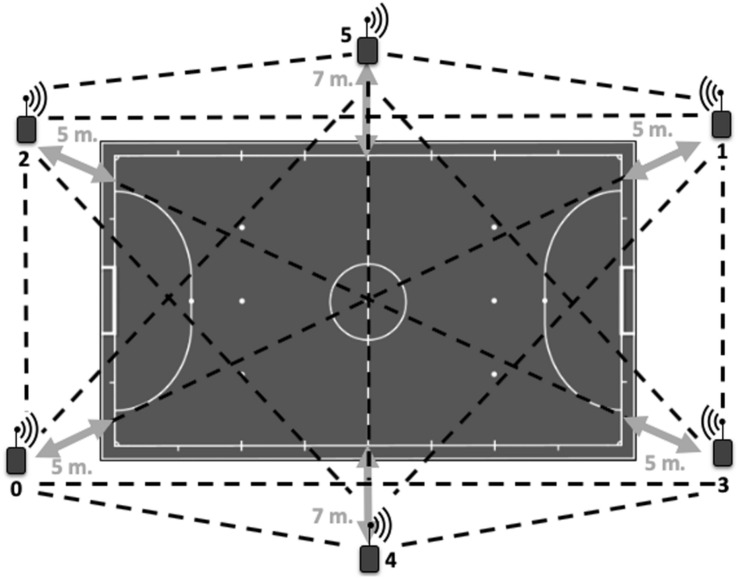
Antennae distribution of Local Positioning System on futsal pitch (Serrano et al., 2020).

The physical activity of each referee during the match was monitored with an individual WIMU PRO^TM^ device (Realtrack Systems S.L., Almería, Spain) with a frequency of 18 Hz was ubicated in a mini pocket of a special vest located between the shoulder blades. The Local Positioning System (LPS) with Ultra-Wide-Band technology (UWB), which was installed on the futsal pitch where the matches were played, it was activated after warm up of referees with an autocalibration of the antennae of 5 min (Bastida-Castillo et al., 2019). This system is constituted by a set of six antennae (Figure 1) that transmit the radiofrequency signal almost under the same principle as the GPS system (Sczyslo et al., 2008). The accuracy and reliability of LPS has been demonstrated in reference to determining distance covered, speed, mean velocity or accelerations for intermittent activities (Stevens et al., 2014; Serpiello et al., 2018). The LPS can calculate the distance or acceleration extracting the means of measuring the changes in the frequency of the periodic signal emitted (Rico-González et al., 2020). Furthermore, the use of device with a 10-Hz sampling frequency, it has been shown that the occurrence of high-intensity accelerations and decelerations can be reliably obtained (Harper et al., 2019). The WIMU PRO^TM^ have showed an accuracy (bias: 0.57–5.85%), test–retest reliability (%TEM: 1.19), and inter-unit reliability (bias: 0.18) in Bastida Castillo et al. (2018) and a large ICC for the x-coordinate (0.65) and a very large ICC for the y-coordinate (0.88), with a good 2%TEM in Bastida-Castillo et al. (2019). The heart rate (HR) was monitored during all games by using a cardiac frequency band (Garmin Ltd., Olathe, KS, United States) synchronized with the inertial device through wireless ANT + technology (García-Santos et al., 2019).

The specific software SPRO^TM^ v. 960 (Realtrack Systems S.L., Almería, Spain) was used to obtain and analyze the referee performance for each match. The physical activity were considered in accordance with previous futsal studies (Ribeiro et al., 2020; Serrano et al., 2020). *The movement pattern variables were*: Total Distance Covered (m); High-Speed Running Distance (HSR: >15.1 km⋅h^–1^); High Speed Running Count (n); High Speed Running Percent (%); Distance-covered in speed zones – ZONE 1 (Z1): walking and low-intensity running (0–10 km⋅h^–1^), ZONE 2 (Z2): medium-intensity running (10.1–15 km⋅h^–1^), ZONE 3 (Z3): high-intensity running (15.1–18 km⋅h^–1^), ZONE 4 (Z4): sprinting (>18.1 km⋅h^–1^); Sprint Distance (m); Sprint Count (n); Sprint Average Duration (s); Maximal Speed (Speed_MAX_: km⋅h^–1^). *The heart rate variables were*: Maximal Heart Rate (HR_MAX_: bpm); Average Heart Rate (HR_AVG_: bpm); Relative HR percentage (HR_RELATIVE_:%). *The acceleration and decelerations variables were*: Explosive Distance (distance with ACC > 1.12 m⋅s^–2^); Total number (n) and distance (m) of Accelerations (ACC: m⋅s^–2^) and Decelerations (DEC: m⋅s^–2^); Maximal Acceleration (ACC_MAX_: m⋅s^–2^) and Deceleration (DEC_MAX_: m⋅s^–2^); Number (n) and distance (m) of accelerations and decelerations for high intensity (>3 m⋅s^–2^).

### Statistical Analysis

Data are presented as means ± standard deviations (SD) along with the 95% confidence interval (95%CI). Before carrying out the analyses Shapiro–Wilk distribution test was performed to confirm a normal distribution of the variables. Differences between first half vs. second half were evaluated through as paired-samples *T*-test procedure. The level of significance was set at *p* < 0.05. The *post hoc* analysis was adjusted using the Bonferroni method. Effect size (ES) was calculated and defined as follows (Cohen, 1992): trivial (ES < 0.19); small (ES = 0.2–0.49); medium (ES = 0.50–0.79) and large (ES > 0.8). All data were statistically analyzed using SPSS V24.0 and G^∗^Power, for Windows.

## Results

Movement pattern and heart rate variables are presented in Table 1. HSR count (n) showed higher values in first half (+3.07; ES: 0.47) than second half. Additionally, in the second half the HR_RELATIVE_ was significantly lower than the first half of the match (−2.46%; ES: 0.29). The rest of variables presented in Table 1 did not revealed significant differences (*p* > 0.05).

**TABLE 1:** Movement pattern and heart rate demands of referees during match.

Movement pattern							

Variables	Full match	First half	Second half	Sig. (*p*)	ES	95% CI	
Total distance (m)	5719.92 ± 249.66	2888.40 ± 122.56	2831.52 ± 150.26	0.08	0.41	−8.61	122.37
HSR distance (m)	442.84 ± 108.87	235.06 ± 67.58	207.78 ± 57.86	0.12	0.44	−9.12	63.69
HSR count (n)	45.36 ± 12.16	24.21 ± 6.65	21.14 ± 6.31*	0.02	0.47	0.47	5.66
HSR distance percent (%)	7.71 ± 1.72	8.12 ± 2.19	7.31 ± 1.90	0.19	0.40	−0.48	2.09
Sprint distance (m)	80.09 ± 39.62	41.55 ± 28.64	38.54 ± 17.76	0.67	0.13	−12.28	18.30
Sprints count (n)	9.00 ± 4.66	4.50 ± 3.13	4.50 ± 2.28	1.00	0.00	−1.66	1.66
Sprint AVG duration (s)	1.85 ± 0.23	1.90 ± 0.28	1.80 ± 0.29	0.29	0.34	−0.10	0.29
Speed_MAX_ (km/h)	19.78 ± 0.58	19.74 ± 0.58	19.70 ± 0.56	0.34	0.07	−0.05	0.13

**Heart rate**							

HR_MAX_ (bpm)	165.77 ± 11.45	164.45 ± 17.43	158.64 ± 12.98	0.13	0.38	−2.04	13.67
HR_AVG_ (bpm)	146.00 ± 16.11	149.36 ± 17.08	142.55 ± 15.92	0.05	0.41	−0.16	13.79
HR_RELATIVE_ (%)	74.68 ± 8.70	74.95 ± 8.68	72.48 ± 8.45*	0.00	0.29	0.77	4.16

*Significant differences between first and second half for a given variable (*p* < 0.05); CI, confidence interval; ES, effect size; HSR, High Speed Running; AVG, average; HR, Heart Rate; MAX, maximal.

**FIGURE 2:**
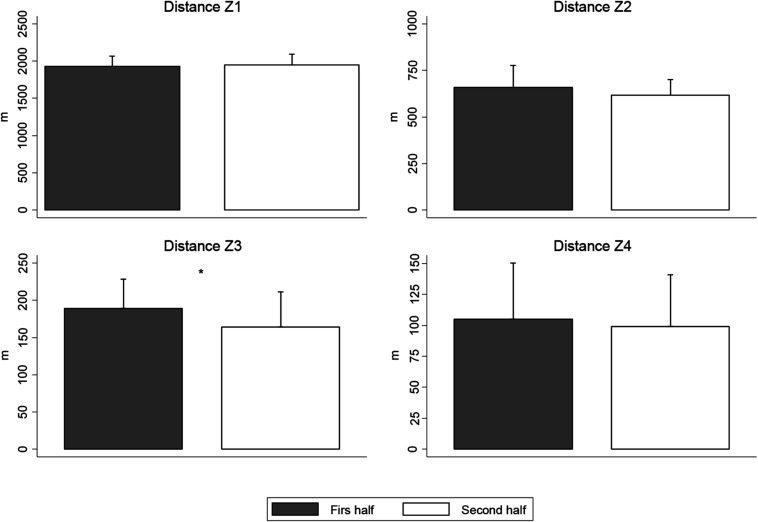
Distance covered in different speed ranges. *Significant differences between first and second half for a given variable (*p* < 0.05). Z1, zone 1 (0–10 km⋅h^–1^); Z2, zone 2 (10.1–15 km⋅h^–1^); Z3, zone 3 (15.1–18 km⋅h^–1^); Z4, zone 4 (>18.1 km⋅h^–1^).

Figure 2 describes the distance in different ranges of speed covered by referees. No significant differences (*p* > 0.05) were revealed for all ranges of speed, except distance covered in Zone 3 (*p* = 0.004). This range was significantly lower in the second period of the match in comparison with the first period (−24.48 m; ES: 0.56).

The acceleration and deceleration variables are shown in Table 2. The referees evidenced a lower explosive distance in the second period compared with the first period (−50.31 m; ES: 0.70). The number of high-intensity acceleration (+10.29; ES: 0.92) and deceleration (+24.86; ES: 0.99) actions revealed significant higher values than those of the second half (*p* < 0.05). Furthermore, distance in ACC and DEC also revealed significant lower results in second half (−40.07 m; ES: 0.63 and −26.81 m; ES: 0.40, respectively) compared to first half.

**TABLE 2:** Acceleration and deceleration demands of referees during match.

Variables	Full match	First half	Second half	Sig. (*p*)	ES	95% CI	
Explosive distance (m)	922.04 ± 137.31	486.17 ± 80.12	435.86 ± 63.99*	0.00	0.70	23.37	77.25
ACC (n)	954.79 ± 191.30	495.57 ± 102.00	459.21 ± 95.74*	0.02	0.37	7.23	65.49
DEC (n)	2096.29 ± 335.81	1045.43 ± 166.41	1050.86 ± 179.28	0.81	0.03	−53.38	42.52
ACC_MAX_ (m⋅s^–2^)	4.56 ± 0.39	4.54 ± 0.39	4.50 ± 0.39	0.14	0.10	−0.01	0.09
DEC_MAX_ (m⋅s^–2^)	−4.10 ± 0.32	−4.09 ± 0.32	−4.08 ± 0.32	0.50	0.03	−0.04	0.02
ACC distance (m)	391.15 ± 114.37	215.61 ± 74.82	175.54 ± 51.82*	0.02	0.63	5.98	74.17
DEC distance (m)	323.51 ± 130.31	175.16 ± 67.78	148.35 ± 66.26*	0.01	0.40	8.66	44.96
ACC > 3 m⋅s^–2^ (n)	80.86 ± 20.01	45.57 ± 13.90	35.29 ± 8.48*	0.01	0.92	3.71	16.86
DEC > 3 m⋅s^–2^ (n)	236.14 ± 45.08	130.50 ± 29.41	105.64 ± 20.37*	0.00	0.99	11.59	38.12
ACC > 3 m⋅s^–2^ (m)	317,8 ± 82.58	171.71 ± 46.53	146.09 ± 44.50*	0.03	0.56	3.48	47.76
DEC > 3 m⋅s^–2^ (m)	284.72 ± 97.57	153.18 ± 50.24	131.54 ± 49.20*	0.00	0.44	10.57	32.73

*Significant differences between first and second half for a given variable (*p* < 0.05); CI, confidence interval; ES, effect size; ACC, acceleration; DEC, deceleration; MAX, maximal.

## Discussion

The purpose of this study was to analyze the activity profile and to compare the physical demands of elite futsal referees between first and second halves during official matches from Spanish Futsal Cup. This is the first research to describe the activity profile of professional futsal referees during an official competition with tracking time-motion technology. The main findings were that high intensity running distance and acceleration decrease during second period of the match. Additionally, the low and moderate intensity running distances and the total number of accelerations remain similar in both periods.

Previous studies have investigated the physical performance of futsal referees (Rebelo et al., 2011; Dixon, 2014; Ahmed et al., 2017). The activity profile revealed by these authors showed similar outcomes to those of the present investigation in relation to volume variables (Rago et al., 2020) corresponding to total distance (5719.91 ± 249.66 m). Additionally, the activity profile of referees presented long distances of slow and moderate speeds (5156.42 ± 456.76 m) among with less distances of high-speed running and sprint (557.37 ± 164.30 m). Although the distribution of activities reported comparable values, the results of the distances covered in the different ranges of speed by the referees of the present manuscript were dissimilar to those of previous studies. It may be explained by the different speed categories selected (Rebelo et al., 2011; Ahmed et al., 2017). Nevertheless, the data obtained in present study confirmed the specific motor pattern of elite futsal referees. Regarding these results, the training programs should be adjusted according to specific competitive characteristics (García-Santos et al., 2019).

The analysis of internal load showed that referees had a HR_AVG_ of 146.00 ± 16.1 and they were working around 75% HR_MAX_. Although, the heart rate could modify due to level of competition or type of referee, similar values to previous investigations due to prior investigations (Rebelo et al., 2011; Dixon, 2014; Ahmed et al., 2017). These outcomes showed that the level of fitness futsal referees must be requirement to tolerate this values. Moreover, the variables of heart rate revealed a reduction in the second half, although without significative differences, it might indicate a tendency to reduce the performance of internal load. This could be influenced by the situational variables of the match (Castillo et al., 2016).

In the comparison of performance between periods of the match, Ahmed et al. (2017) reported a decline in total distance covered by futsal Iraq Futsal Premier League referees (3093 ± 271 vs. 2850 ± 219 m). Contrary to the findings of these studies, the present results showed similar total distances covered in both halves (2888.39 ± 122.55 vs. 2831.51 ± 150.26 m). The movement patterns of referees were monitored by new tracking device in the present study. The difference in the technology or the type of competition studied might explain why the results were not similar. There are investigations with analyses of the relative distance covered by elite futsal players during official matches, which demonstrated that they maintain similar values between halves (Serrano et al., 2020) or even experiment an increase (Ribeiro et al., 2020). The development of the game and the different contextual variables that exist during matches could be the reason of these differences (Méndez et al., 2019). This fact could be influenced by the referee having to be near the game, which means they need to cover different distances in each game depending the course of the match.

The results from the distance covered and the number of high-speed runs in the present investigation revealed a significant decrease in the second half compared with the first half. The performance of this variable have showed similar behaviors that others futsal referees studies in the comparison between halves (Rebelo et al., 2011; Ahmed et al., 2017). The specific situations of futsal matches might reduce the rate of the play in the second half affecting the referees’ performance in terms of these types of physical demands. Although the goalkeeper-player situations did not contemplate in the present investigation, this tactic situation is preferentially adopted in several futsal games (Méndez-Domínguez et al., 2019) and it could modify the speed of the play and that to influence the referee’s activity. The high speed performance of the present results showed lower total distance of high-speed runs (442.84 ± 108.87) than previous investigations (Rebelo et al., 2011; Ahmed et al., 2017). The zones of speed used and the tracking technology might explain the differences distance covered in this manuscript compared to previous studies. Moreover, the type of competition analyzed in the present study was different format than the other studies which were regular competition. The studies with elite futsal players of different leagues have exposed dissimilar physical performances in this type of demands (De Oliveira Bueno et al., 2014; Ribeiro et al., 2020; Serrano et al., 2020) and it could happen in referees too.

With regard to the acceleration and deceleration variables, this study is the first to provide detailed information on these kinds of actions by futsal referees. Previous research have reported different actions, such as stops, turns, or sideways running during games (Rebelo et al., 2011; Ahmed et al., 2017). The results of the present manuscript confirm that this type of demands are an important part of the activity profile of futsal referees due to elevated number of high-intensity accelerations and decelerations. Recent futsal studies have showed that these demands should be considered in profile of player because the ability to accelerate and decelerate have been evidenced during games (Illa et al., 2020; Ribeiro et al., 2020; Serrano et al., 2020). Additionally, the values of high-intensity accelerations and decelerations indicated a decrease of the performance in the second half. Although, the present study did not analyze the performance of players, the development of game and activity of players be able to influence the performance of these demands due to the activity of referees is dictated by the activity of the game (Ahmed et al., 2017). This type of actions require high eccentric force and it might produce an accumulation of muscular fatigue (Edwards, 2018). Therefore, the monitoring of this actions revealed some interesting outcomes which could be helpful in the design of strength and conditioning programs of futsal referees.

## Limitations

One of the limitations this study is the low number of referees and matches assessed. However, this research analyzed the whole Spanish Futsal Cup 2020 and tested the highest-level referees in Spain. Another limitation is the tournament itself as only winners moved to the following round and it could have influence over the performance of matches. Therefore, future studies should analyze longer tournaments like regular league and higher number of referees to confirm our findings. Additionally, the possible correlation between the physical parameters of referees and players’ physical demands in competitions could lend support to understanding the physical profile and performance of the referees (Ahmed et al., 2017; García-Santos et al., 2020). Furthermore, the knowledge of this relationship could help to detect the possibility of most demanding scenarios from referees during the competition (Gabbett, 2016; Oliva-Lozano et al., 2020; Vázquez-Guerrero et al., 2020).

## Conclusion and Practical Applications

Thus, the findings confirmed that the specific profile activity of elite futsal referees show long distances of slow and moderate speeds with minor high-speed running and sprint distances. Moreover, a great number of high-intensity deceleration actions were proved. In addition, the present study found a decrease in physical performance by the referees in relation to the high-intensity actions when comparing the first and second halves. It could be due to the referees must be follow the players and the specific situations during futsal games. It might reduce the intensity of the match in the second half affecting the referees’ performance.

Finally, the use of the tracking device to monitoring physical performance provides knowledge of the specific activity profile from futsal referees. This information to can be useful to design new physical testing and more accurate the training programs. This will help the referees to meet the workload demand during the game and to make accurate decisions without a high level of fatigue. Therefore, the possibility of associating the acceleration and deceleration variables among specific movements of referees may contribute to producing individual training programs to improve this kind of skill and to reduce the injury risk due to these physical demands.

## Data Availability Statement

The data analyzed in this study is subject to the following licenses/restrictions: The raw data supporting the conclusions cannot be made available due to the restrictions defined by the Spanish National Committee of Referees. Requests to access these datasets should be directed to CS, carlos.serrano.90@hotmail.com.

## Ethics Statement

The studies involving human participants were reviewed and approved by the Ethics Committee of the European University of Madrid (CIPI35/2019). The patients/participants provided their written informed consent to participate in this study.

## Author Contributions

CS and JS-S: conceptualization and investigation. CS and JF: methodology. CS, JF, JS-S, and JG-U: writing and original draft preparation. LG and EH: writing, review, and editing. All authors have read and agreed to the published version of the manuscript.

## Conflict of Interest

The authors declare that the research was conducted in the absence of any commercial or financial relationships that could be construed as a potential conflict of interest.
